# Temporal changes in the clinical-epidemiological profile of patients with Chagas disease at a referral center in Brazil

**DOI:** 10.1590/0037-8682-0040-2021

**Published:** 2021-06-02

**Authors:** Alejandro Marcel Hasslocher-Moreno, Roberto Magalhaes Saraiva, Pedro Emmanuel Alvarenga Americano do Brasil, Luiz Henrique Conde Sangenis, Sergio Salles Xavier, Andréa Silvestre de Sousa, Gilberto Marcelo Sperandio-da-Silva, Fernanda de Souza Nogueira Sardinha Mendes, Andréa Rodrigues da Costa, Marcelo Teixeira de Holanda, Henrique Horta Veloso, Flavia Mazzoli-Rocha, Fernanda Martins Carneiro, Luciana Fernandes Portela, Mauro Felippe Felix Mediano

**Affiliations:** 1 Fundação Oswaldo Cruz, Instituto Nacional de Infectologia Evandro Chagas, Rio de Janeiro, RJ, Brasil.; 2 Universidade Federal do Rio de Janeiro, Faculdade de Medicina, Rio de Janeiro, RJ, Brasil.

**Keywords:** Chagas disease, Epidemiologic studies, Cohort studies

## Abstract

**INTRODUCTION:**

We aimed to describe the sociodemographic, epidemiological, and clinical characteristics of patients with chronic Chagas disease (CD) at an infectious disease referral center. Changes in patient profiles over time were also evaluated.

**METHODS:**

This retrospective study included patients with CD from November 1986-December 2019. All patients underwent an evaluation protocol that included sociodemographic profile; epidemiological history; anamnesis; and physical, cardiologic, and digestive examinations. Trend differences for each 5-year period from 1986 to 2019 were tested using a nonparametric trend test for continuous and generalized linear models with binomial distribution for categorical variables.

**RESULTS:**

A total of 2,168 patients (52.2% women) were included, with a mean age of 47.8 years old. White patients with low levels of education predominated. The reported transmission mode was vectorial in 90.2% of cases. The majority came from areas with a high prevalence (52.2%) and morbidity (67.8%) of CD. The most common clinical presentation was the indeterminate form (44.9%). The number of patients referred gradually decreased and the age at admission increased during the study period, as did the patients’ levels of education.

**CONCLUSIONS:**

The clinical profile of CD is characterized by a predominance of the indeterminate form of the disease. Regarding the patients who were followed up at the referral center, there was a progressive increase in the mean age and a concomitant decrease in the number of new patients. This reflects the successful control of vector and transfusion transmission in Brazil as well as the aging population of patients with CD.

## INTRODUCTION

Chagas disease (CD) is considered a neglected tropical disease by the World Health Organization, with an estimated 6-7 million people infected worldwide. The implementation of CD vector and transfusion control programs in the 1980s significantly decreased the rate of disease transmission in Latin American countries. However, several challenges have hampered the effective implementation of disease surveillance due to new outbreaks of orally transmitted CD in endemic countries and the possibility of vertical transmission even in nonendemic areas. Integrated surveillance and healthcare interventions are now directed at a large contingent of patients already infected with *Trypanosoma cruzi* (*T. cruzi*), a significant portion of whom may develop chronic Chagas heart disease, a major determinant of morbidity and mortality. Owing to rapid globalization, CD cases are no longer restricted to Latin America, constituting a new challenge in the battle against this disease^1^. In Brazil, the process of CD urbanization in the last decades of the 20th century has increased the number of patients with CD in urban cities, which has increased the demand for local health care services. This new urban context has also prompted the modification of the clinical-epidemiological profile of patients with CD, evidenced by changes in work activities, food consumption patterns, increased age, significant prevalence of comorbidities, and social determinants as a whole^2^ ^-^ ^4^. 

 The first national serological survey that evaluated the prevalence of CD in Brazil aimed to quantify the endemic transmission of CD^5^. This study was conducted in rural areas of Brazil between 1975 and 1980, with an estimated national seroprevalence CD rate of 4.22%. A second national serological CD survey^6^ conducted between 2001 and 2008 analyzed the seroprevalence in children aged up to five years, which only reported a CD serum positivity rate of 0.03%. The later survey highlighted the impact of the CD control measures implemented in previous decades, which led the Pan American Health Organization to grant Brazil an International Certificate of Elimination of CD transmission by *Triatoma infestans* and blood transfusions^7^. In Brazil, the current estimated prevalence of CD is much lower than that reported in the 1970s. However, the estimates reported in previous studies on the prevalence of CD are imprecise and subject to criticism due to the lack of standardized data collection and heterogeneity in most of these estimates^8^. Therefore, at present, the exact number of Brazilians with CD is unknown, although it is projected to be between 1 and 1.5 million people^9^. Currently, new cases of CD in Brazil are mostly restricted to the Legal Amazon, with oral being the primary route of transmission, particularly through the consumption of a local fruit called *açaí* when it is contaminated with *T. cruzi* ^10^.

With globalization, the disease has spread to countries in the Northern Hemisphere, particularly in the United States and Spain^11^. Because of this new geographic rearrangement of CD, studies describing the clinical and epidemiological profile of patients with chronic CD have been conducted in urban healthcare facilities in both endemic^12^ ^-^ ^15^ and nonendemic regions^16^ ^-^ ^19^. Some of these facilities have also become reference centers for the treatment of CD that offer specialized care^20^ ^-^ ^23^.

The Evandro Chagas Institute of Infectious Diseases of the Oswaldo Cruz Foundation (INI-Fiocruz), located in Rio de Janeiro, Brazil, is a national reference center for the treatment and research of infectious and tropical diseases covered by the *Sistema Único de Saúde* (*SUS*), the Brazilian National Health Service. INI-Fiocruz receives patients from various regions of the country and offers comprehensive and multidisciplinary care to patients with CD. To date, few studies have addressed the clinical and epidemiological profiles of the Brazilian population with chronic CD. The present study aimed to describe the clinical and epidemiological profiles of a historical cohort of patients with chronic CD followed up at the INI-Fiocruz.

## METHODS

This was a retrospective descriptive study including patients diagnosed with chronic CD who were referred to the outpatient center of the INI-Fiocruz between November 1986 and December 2019. Clinical and epidemiological data were retrieved from the medical records.

After the serological diagnosis of chronic CD was confirmed by two simultaneous reactive serological techniques, all patients underwent an initial evaluation protocol, which included sociodemographic information (age, level of education, and race); epidemiological history (transmission mode, country and state of origin, time away from endemic area); clinical anamnesis; a physical examination focused on chronic CD-related cardiovascular and digestive signs and symptoms; a 12-lead electrocardiogram (ECG); and a two-dimensional Doppler echocardiogram. According to the presence of symptoms related to the digestive form of CD, the following examinations were performed: upper gastrointestinal endoscopy, esophagography, colonoscopy, and a contrast barium enema. The level of education was categorized based on the number of years of formal study as illiterate, < 9 years, or > 9 years. Race was self-reported and classified as white, black, mulatto, or indigenous.

Clinical forms of chronic CD were retrospectively classified according to the 2nd Brazilian Consensus on Chagas Disease^24^. The cardiac form was classified into A, B1, B2, C, or D stages, and the digestive form was classified into megaesophagus, megacolon, or both megaesophagus and megacolon. Information about the region of origin was classified according to the prevalence and morbidity of chronic CD, which was based on serological data from national prevalence surveys^5^ and a national electrocardiographic survey^25^. The prevalence was categorized as low (< 2%), medium (2%-4%), high (> 4%), and nonendemic (Rio de Janeiro and Espírito Santo), whereas the levels of morbidity by area were categorized as low (normal ECGs > 50%), high (normal ECGs < 50%), and nonendemic areas (Rio de Janeiro and Espírito Santo). 

### Data analysis

Descriptive statistics are presented as means (standard deviations) for continuous and absolute frequencies (percentages) for categorical variables. Trend differences for each 5-year period from 1986 to 2019 were tested using a nonparametric trend test for continuous (*nptrend* command in Stata 13.0) variables and using generalized linear models with binomial distribution for categorical variables (*binreg* command in Stata 13.0). The independent variable was the 5-year period, and the dependent variables were the binary classes of each categorical variable. The link choice was selected according to the lowest Bayesian information criterion. Statistical significance was set at a 2-tailed p-value of <0.05. All statistical analyses were performed using Stata software (version 13.0; StataCorp LP.; College Station, TX, USA). 

### Ethics approval

This study was approved by the INI-Fiocruz Research Ethics Committee (number CAAE:35748820.1.0000.5262) on September 2, 2020 and was carried out in accordance with the 1964 Declaration of Helsinki and its later amendments. The need for informed consent was waived considering the retrospective nature of the study.

## RESULTS

The characteristics of the patients referred to the INI-Fiocruz are shown in Table 1. A total of 2,168 patients (52.2% women) were included from August 1986 to December 2019, with a mean age of 47.8 years (range, 13-88 years). The plurality self-reported as white (49.8%) and had < 9 years of education (80.5%). The reported transmission mode was vectorial in 90.2% of the patients. The majority, originating from Brazil (98.7%), were born in areas with high prevalence (52.2%) and morbidity (67.8%) of CD, mostly Minas Gerais and Bahia, and had moved away from endemic areas for >20 years (65.8%). The indeterminate form was the most common clinical presentation (44.9%), and stage A (21.9%) was the most common clinical presentation of the cardiac form. Only a minority of patients had digestive or cardiodigestive forms (11.7%), with 7.4% presenting with megaesophagus, 2.5% with megacolon, and 1.8% with both megaesophagus and megacolon.

**TABLE 1:** Characteristics of patients admitted at the INI-Fiocruz (n=2,168).

Variable	Mean (SD) Minimum-
	Maximum or Frequency (%)
Age (years)	47.8 (12.8) 13-88
Female sex	1,132 (52.2)
Race	
White	1,080 (49.8)
Mulatto	815 (37.6)
Black	261 (12.0)
Indigenous	12 (0.6)
Level of education	
Illiterate	448 (20.7)
< 9 years	1,297 (59.8)
> 9 years	423 (19.5)
Transmission mode	
Vectorial	1,956 (90.2)
Transfusion	124 (5.7)
Vertical	62 (2.9)
Oral	2 (0.1)
Not identified	24 (1.1)
Country of origin	
Brazil	2,140 (98.7)
Other Latin American countries	28 (1.3)
Region of origin according to prevalence	
Nonendemic Chagas disease	102 (4.7)
Low Chagas disease prevalence	260 (12.0)
Medium Chagas disease prevalence	674 (31.1)
High Chagas disease prevalence	1,132 (52.2)
Region of origin according to morbidity	
Nonendemic Chagas disease	102 (4.7)
Low Chagas disease morbidity	596 (27.5)
High Chagas disease morbidity	1,470 (67.8)
Time away from endemic area	
None	82 (3.8)
1 to 20 years	577 (26.6)
>20 years	1,426 (65.8)
Nonendemic area	83 (3.8)
Clinical form (n=2,136)	
Indeterminate	960 (44.9)
Cardiac	924 (43.3)
Digestive	125 (5.9)
Cardiodigestive	127 (5.9)
Clinical cardiac stages^a^ (n=2,136)	
None	1085 (50.8)
Stage A	467 (21.9)
Stage B1	250 (11.7)
Stage B2	108 (5.1)
Stage C	206 (9.6)
Stage D	20 (0.9)
Clinical digestive presentation	
None	1914 (88.3)
Megaesophagus	160 (7.4)
Megacolon	54 (2.5)
Megaesophagus and megacolon	40 (1.8)

^a^According to the 2^nd^ Brazilian Consensus on Chagas Disease^24^. Abbreviations: **INI-Fiocruz**: The Evandro Chagas Institute of Infectious Diseases of the Oswaldo Cruz Foundation; **SD:** standard deviation.

The characteristics of patients referred to the INI-Fiocruz for each 5-year period are shown in Table 2. The number of patients referred increased from the 1986-1990 period (n=138) to the 2000-2004 period (n=569) and then gradually decreased until the 2015-2019 period (n=162). The age at the time of referral increased during all study periods (p<0.001). The number of white patients decreased over time (p<0.001), whereas the number of mulatto (p<0.001) and black (p<0.001) patients increased. Illiterate patients decreased over time (p =0.02), and those with >9 years of education increased (p=0.008). The vectorial and oral transmission modes increased over the study period (p<0.001 for both) while transfusion transmission decreased (p<0.001).

**TABLE 2:** Characteristics of patients admitted at the INI-Fiocruz for each 5-year period (n=2,168)

Variable	Mean (SD) inimum-Maximum or Frequency (%)							p-value for trend
	1986-1989	1990-1994	1995-1999	2000-2004	2005-2009	2010-2014	2015-2019	
	(n=138)	(n=295)	(n=498)	(n=569)	(n=307)	(n=199)	(n=162)	
Age (years)	44.3 (10.6)	45.3 (12.0)	45.9 (11.3)	46.3 (12.2)	49.7 (14.1)	53.5 (12.9)	57.1 (12.9)	<0.001
	22-73	19-85	16-84	13-84	15-87	19-85	21-88	
Female sex	71 (51.8)	168 (57.0)	252 (50.60)	283 (49.7)	158 (51.5)	116 (58.3)	84 (51.9)	0.93
Race								
White	76 (55.1)	174 (59.0)	244 (49.0)	314 (55.2)	154 (50.2)	82 (41.2)	36 (22.2)	<0.001
Mulatto	52 (37.7)	91 (30.9)	197 (39.6)	180 (31.6)	101 (32.9)	97 (48.7)	97 (59.9)	<0.001
Black	8 (5.8)	28 (9.5)	52 (10.4)	72 (12.7)	52 (16.9)	20 (10.1)	29 (17.9)	<0.001
Indigenous	2 (1.5)	2 (0.7)	5 (1.0)	3 (0.5)	0 (0.0)	0 (0.0)	0 (0.0)	0.02
Level of education								
Illiterate	32 (23.2)	52 (17.6)	141 (28.3)	110 (19.3)	50 (16.3)	34 (17.1)	29 (17.9)	0.02
< 9 years	86 (62.3)	182 (61.7)	268 (53.8)	350 (61.5)	209 (68.1)	117 (58.8)	85 (52.2)	0.87
> 9 years	20 (14.5)	61 (20.7)	89 (17.9)	109 (19.2)	48 (15.6)	48 (24.1)	48 (29.6)	0.008
Transmission mode								
Vectorial	119 (86.2)	254 (86.1)	435 (87.4)	525 (92.3)	285 (92.8)	189 (95.0)	149 (92.0)	<0.001
Transfusion	16 (11.6)	29 (9.8)	45 (9.0)	16 (2.8)	8 (2.6)	3 (1.5)	7 (4.3)	<0.001
Vertical	1 (0.7)	8 (2.7)	13 (2.6)	24 (4.2)	9 (2.9)	4 (2.0)	3 (1.9)	0.81
Oral	0 (0.0)	0 (0.0)	0 (0.0)	0 (0.0)	0 (0.0)	0 (0.0)	2 (1.2)	<0.001
Not identified	2 (1.5)	4 (1.4)	5 (1.0)	4 (0.7)	5 (1.6)	3 (1.5)	1 (0.6)	0.84
Country of origin								
Brazil	135 (97.8)	289 (98.0)	491 (98.6)	565 (99.3)	302 (98.4)	197 (99.0)	161 (99.4)	0.15
Other Latin American countries	3 (2.2)	6 (2.0)	7 (1.4)	4 (0.7)	5 (1.6)	2 (1.0)	1 (0.6)	
Region of origin according to prevalence								
Nonendemic Chagas disease	5 (3.6)	18 (6.1)	19 (3.8)	31 (5.5)	14 (4.6)	8 (4.0)	7 (4.3)	0.78
Low Chagas disease prevalence	7 (5.1)	17 (5.8)	58 (11.7)	74 (13.0)	42 (13.7)	38 (19.1)	24 (14.8)	<0.001
Medium Chagas disease prevalence	37 (26.8)	93 (31.5)	137 (27.5)	186 (32.7)	101 (32.9)	56 (28.1)	64 (39.5)	0.04
High Chagas disease prevalence	89 (64.5)	167 (56.6)	284 (57.0)	278 (48.9)	150 (48.9)	97 (48.7)	67 (41.4)	<0.001
Region of origin according to morbidity								
Nonendemic Chagas disease area	5 (3.7)	18 (6.1)	19 (3.8)	31 (5.5)	14 (4.6)	8 (4.0)	7 (4.3)	0.78
Low Chagas disease morbidity area	22 (15.9)	67 (22.7)	130 (26.1)	159 (27.9)	95 (30.9)	64 (32.2)	59 (36.4)	<0.001
High Chagas disease morbidity area	111 (80.4)	210 (71.2)	349 (70.1)	379 (66.6)	198 (64.5)	127 (63.8)	96 (59.3)	<0.001
Time away from endemic area								
None	1 (0.7)	8 (2.7)	29 (5.8)	19 (3.3)	16 (5.2)	5 (2.5)	4 (2.5)	0.90
1 to 20 years	16 (11.6)	57 (19.3)	129 (25.9)	179 (31.5)	108 (35.2)	62 (31.2)	26 (16.1)	0.002
>20 years	117 (84.8)	216 (73.2)	323 (64.9)	346 (60.8)	173 (56.4)	124 (62.3)	127 (78.4)	0.005
Nonendemic area	4 (2.9)	14 (4.8)	17 (3.4)	25 (4.4)	10 (3.3)	8 (4.0)	5 (3.1)	0.77
Clinical form (n=2,136)								
Indeterminate	56 (40.6)	135 (45.8)	264 (53.0)	280 (51.2)	107 (35.9)	66 (33.3)	52 (32.1)	<0.001
Cardiac	68 (49.3)	142 (48.1)	204 (41.0)	221 (40.4)	140 (47.0)	81 (40.9)	68 (42.0)	0.20
Digestive	6 (4.3)	12 (4.1)	14 (2.8)	26 (4.8)	23 (7.7)	28 (14.2)	16 (9.9)	<0.001
Cardiodigestive	8 (5.8)	6 (2.0)	16 (3.2)	20 (3.6)	28 (9.4)	23 (11.6)	26 (16.0)	<0.001
Clinical cardiac stages^a^ (n=2,136)								
Stage A	36 (26.1)	58 (19.7)	104 (20.9)	129 (23.6)	66 (22.2)	33 (16.7)	41 (25.3)	0.93
Stage B1	17 (12.3)	45 (15.3)	48 (9.6)	48 (8.8)	40 (13.4)	32 (16.2)	20 (12.4)	0.59
Stage B2	13 (9.4)	16 (5.4)	26 (5.2)	21 (3.8)	17 (5.7)	8 (4.0)	7 (4.3)	0.10
Stage C	9 (6.5)	21 (7.1)	39 (7.8)	38 (8.0)	44 (14.8)	30 (15.2)	25 (15.3)	<0.001
Stage D	1 (0.7)	8 (2.7)	3 (0.6)	5 (0.9)	1 (0.3)	1 (0.5)	1 (0.6)	0.07
Clinical digestive presentation								
Megaesophagus	8 (5.8)	14 (4.8)	19 (3.8)	26 (4.6)	35 (11.4)	33 (16.6)	25 (15.4)	<0.001
Megacolon	3 (2.2)	3 (1.0)	8 (1.6)	12 (2.1)	7 (2.3)	10 (5.0)	11 (6.8)	<0.001
Megaesophagus and megacolon	3 (2.2)	1 (0.3)	3 (0.6)	10 (1.8)	9 (2.9)	8 (4.0)	6 (3.7)	0.001

^a^According to the 2^nd^ Brazilian Consensus on Chagas Disease (2015)^24^. Abbreviations: **INI-Fiocruz**: The Evandro Chagas Institute of Infectious Diseases of the Oswaldo Cruz Foundation; **SD:** standard deviation.

The percentage of patients that originated from low CD prevalence areas increased over the study period (p<0.001), while patients originating from high CD prevalence areas decreased (p<0.001). The same pattern was observed for the CD morbidity areas, with an increase in the percentage of patients who originated from low CD morbidity areas (p<0.001) and a decrease in patients from high CD morbidity areas (p<0.001). The number of patients who had moved away from an endemic area for <20 years increased (p=0.002) with a concomitant decrease of patients that had moved away for >20 years (p=0.005).

The clinical presentation characteristics also changed over the study period, with an increase in the digestive form (p<0.001) and a decrease in the indeterminate form (p<0.001). Considering the clinical cardiac stages, there was an increase in the percentage of patients admitted with stage C (p<0.001), however there were no changes observed in the other stages. 

## DISCUSSION

The INI-Fiocruz is a reference center for CD that provides diagnostic interpretation for patients referred from blood banks, primary and secondary care units, private health services, or by spontaneous demand, and it offers integral and multidisciplinary clinical care to patients with CD. In the present study, most patients were long-time residents of the metropolitan region of the state of Rio de Janeiro and who had been away from endemic areas for many years. Although patients lived in Rio de Janeiro, most were migrants from 19 Brazilian states, predominantly from Bahia and Minas Gerais, which constitutes almost 50% of the studied cohort. A study conducted in the 1960s that evaluated natural-born Brazilian citizens with chronic CD residing in the city of Rio de Janeiro reported the same prevalence^26^. A similar profile was reported in a study published by Ianni^27^ of patients living in the city of São Paulo, indicating the migratory profile of the CD-infected population. Dias et al.^28^ studied the status of chronic CD in the Northeastern region and reported that the state of Bahia had the highest prevalence of the disease in the region, which is similar to that of the state of Minas Gerais. They also discussed the social causes of the strong emigration to large urban centers in the Southeastern region, which justifies the significant number of people from Minas Gerais and Bahia in the present cohort. Besides, 1.3% of the patients come from other South American countries, predominantly from Bolivia, a country with a prevalence rate of 4.4% for chronic CD among migrants^29^.

The vector route is the most probable transmission mechanism. The majority of patients reported living in rural areas in houses made of mud and straw and were aware of the triatomine bug, while some even reported intradomicile coexistence with the insect, yet only a few remembered that they had been bitten. Most patients claimed that they were unaware that the triatomine bug was a health risk. This finding indicates the knowledge deficit regarding CD among these patients, which reinforces why most of them do not consider living with triatomine bugs as a threat to their health^30^. Several studies on transmission mechanisms have also reported the vector route as the primary route of disease transmission^12^ ^,^ ^13^ ^,^ ^20^.

This cohort showed a slight predominance of women (52.2%), characterizing a balanced cohort in terms of sex and reflecting the distribution of men and women in the Brazilian population, which is 51% women and 49% men according to the Brazilian Institute of Geography and Statistics. In previous studies, women accounted for 46% to 84% of the total study population^13^ ^-^ ^17^ ^,^ ^20^ ^-^ ^23^ ^,^ ^34^ ^,^ ^36^ ^,^ ^37^.

In the center where this study was conducted, white patients predominated (49.8%). Gontijo et al.^20^ reported the predominance of mulattos (43%) in Minas Gerais, while Gasparim et al.^13^ reported 51.1% of whites in Paraná, suggesting ethnic differences by geographic location. Most patients (59.8%) had low levels of education. This is attributed to the origin of the patients, usually born in rural areas without access to formal education. Gontijo et al.^20^ showed that 84% of patients examined in a reference outpatient clinic in Belo Horizonte, Brazil were either illiterate or semi-illiterate. Pereira et al.^14^ reported that 40.2% of patients in a reference center in Fortaleza were illiterate. The number of white patients decreased over time, whereas the number of mulatto and black patients increased. The number of illiterate patients decreased and those with >9 years of education increased. These data suggest that the Brazilian population of low socioeconomic strata has gained access to both health services and formal education in the last few decades.

The mean age of the patients was 47.8 years old. Field studies performed between 1960 and the early 2000s reported a progressive increase in the mean age of patients with chronic CD over time, from less than 25 to 45 years^31^ ^-^ ^33^. Studies on the clinical epidemiological profile of patients with chronic CD in urban centers reported that the mean age showed the same increasing trend in the last decades, ranging from 37.7 to 67.5 years^12^ ^,^ ^13^ ^,^ ^15^ ^,^ ^20^ ^-^ ^22^. Studies conducted in nonendemic regions of the Northern Hemisphere, where patients migrated from South America, especially Bolivia and Central America, reported younger patients with chronic CD with a mean age ranging from 28.5 to 47 years^16^ ^-^ ^18^ ^,^ ^34^ ^-^ ^37^, reflecting the lack of CD vectorial transmission control in their countries of origin and that CD vectorial transmission remained active in rural areas. The present study showed a progressive increase over time in the age of patients who were included in the study cohort during the 5-year intervals of the study period, with the mean age increasing from 44.3 to 57.1 years. Following the increase in the age of patients with CD, there was an inverse decrease in the number of new patients requesting care at the INI-Fiocruz. An ascending temporal curve was observed between 1986 and 1994, stabilizing between 1995 and 2004, and decreasing in 2005. This behavior may reflect the successful control of vector transmission by *Triatoma infestans* as well as by blood transfusions in Brazil; however this may also indicate the decrease in migration from rural to urban areas verified in recent decades due to the decreased economic power of urban cities consequently attracting fewer people.

Studies on the clinical form of CD conducted in urban areas have shown that the cardiac form predominates, with a prevalence ranging from 56% to 66%^12^ ^-^ ^16^ ^,^ ^22^ ^,^ ^23^ Few studies have reported a higher incidence of the indeterminate form, varying between 56% and 81.6%^17^ ^,^ ^20^. These differences in the prevalence of clinical forms are attributed to the profile of the healthcare unit. CD reference centers usually receive asymptomatic donors from blood banks, asymptomatic family members of patients under follow-up at the institution, and spontaneous demand for serological diagnosis, which tends to mirror the epidemiological reality of the disease in which the indeterminate forms predominate. Symptomatic patients are expected in secondary and tertiary care units, which involve the management of patients with more complex cases. Therefore, cases of the cardiac and digestive forms are more prevalent in these units. In the present study, the indeterminate form was the most prevalent (44.9%). The digestive form, either isolated or associated with heart disease, had a prevalence of 11.8%, which is within the range of the mean prevalence of this clinical form (9%-41%) presented in other studies^12^ ^-^ ^15^ ^,^ ^17^ ^,^ ^19^ ^,^ ^20^ ^,^ ^22^. Regarding the cardiac and cardiodigestive forms, the most common cardiac stage was stage A (44.4%), which, if associated with the indeterminate form, would account for 65.8% of the cohort, with normal left ventricular systolic function on the ECG indicating patients with long-term benign prognoses.

In this cohort, although the indeterminate form was common among all patients throughout the study period, it occurred mainly between 1995 and 2004. Before and after this period, the cardiac form was more prevalent in newly diagnosed patients. Until 1994, the cardiac form reflected patients coming from areas with active vector transmission and with higher CD prevalence and morbidity. After the mid-2000s, although there was an increased number of patients coming from areas of lower CD morbidity, they tended to be older with several comorbidities such as systemic arterial hypertension, dyslipidemia, diabetes mellitus, and ischemic heart disease. These comorbidities usually lead to cardiac changes that are reflected in the ECG and may mimic the electrocardiographic changes of Chagas heart disease leading patients to be classified as having the cardiac form of the disease^38^. The progressive increase in the presence of mega forms of CD over time may also be related to the aging patients in the CD cohort justified by the gradual progression of neuronal degeneration associated with the disease^39^. 

The INI-Fiocruz cohort is mostly comprised of patients who migrated from various Brazilian regions, which may partially increase the representativeness of the total CD-infected population in Brazil, characterized by a predominance of the indeterminate clinical form of the disease. In addition, the progressive decrease in the number of new patients who entered the cohort over the past few years possibly reflects the success of the vector transmission control by *Triatoma infestans* and by the transfusion route in recent decades in Brazil. We also observed the gradual aging of patients with chronic CD. 
